# The Inhibitory Effect of (−)-Epigallocatechin-3-Gallate on Breast Cancer Progression via Reducing *SCUBE2* Methylation and DNMT Activity

**DOI:** 10.3390/molecules24162899

**Published:** 2019-08-09

**Authors:** Jie Sheng, Weilin Shi, Hui Guo, Wenlin Long, Yuxin Wang, Jiangfa Qi, Jinbiao Liu, Yao Xu

**Affiliations:** 1 Institute of Biology and Medicine, College of Life Sciences and Health, Wuhan University of Science and Technology, Wuhan 430081, China; 2 Institute of Medical Microbiology, Jinan University, Guangzhou 510632, China

**Keywords:** EGCG, *SCUBE2* gene, methylation, migration and invasion, breast cancer

## Abstract

Epigenetic modifications are important mechanisms responsible for cancer progression. Accumulating data suggest that (−)-epigallocatechin-3-gallate (EGCG), the most abundant catechin of green tea, may hamper carcinogenesis by targeting epigenetic alterations. We found that signal peptide-CUB (complement protein C1r/C1s, Uegf, and Bmp1)-EGF (epidermal growth factor) domain-containing protein 2 (*SCUBE2*), a tumor suppressor gene, was hypermethylated in breast tumors. However, it is unknown whether EGCG regulates *SCUBE2* methylation, and the mechanisms remain undefined. This study was designed to investigate the effect of EGCG on *SCUBE2* methylation in breast cancer cells. We reveal that EGCG possesses a significantly inhibitory effect on cell viability in a dose- and time-dependent manner and presents more effects than other catechins. EGCG treatment resulted in enhancement of the *SCUBE2* gene, along with elevated E-cadherin and decreased vimentin expression, leading to significant suppression of cell migration and invasion. The inhibitory effect of EGCG on *SCUBE2* knock-down cells was remarkably alleviated. Further study demonstrated that EGCG significantly decreased the *SCUBE2* methylation status by reducing DNA methyltransferase (DNMT) expression and activity. In summary, this study reported for the first time that *SCUBE2* methylation can be reversed by EGCG treatment, finally resulting in the inhibition of breast cancer progression. These results suggest the epigenetic role of EGCG and its potential implication in breast cancer therapy.

## 1. Introduction

Carcinogenesis is a multistage event consisting of initiation, promotion, progression, and malignant conversion phases. Burgeoning evidence has confirmed that the carcinogenesis process not only depends on heritable variability but also on epigenetic modifications, which are correlated with the different genetic code profiles without sequence alterations [1,2]. DNA methylation is one of the epigenetic mechanisms that occurs most commonly with silencing of tumor suppressor genes (TSGs). The altered TSG expression subsequently leads to accumulated physiological changes, paving the way for tumorigenesis [3,4]. A previous study reported that many TSGs are inactivated during neoplastic initiation and progression due to the promoter hypermethylation [5]. Therefore, to reduce methylated state of the TSGs represents an emerging perspective approach for cancer therapies.

DNA methylation is commonly related to increased expression levels or gain-of-function of DNA methyltransferase (DNMT) that initiates the methylation at the 5′-cytosine of the CpG island [6]. The inhibition of DNMT activity would block the DNA strand hypermethylation, resulting in the reversal of the modification level and activation of the silenced genes [7]. Thus, exploring and application of the DNMT inhibitors become promising anticancer modalities. The available inhibitors, such as 5-aza-2′-deoxycytidine and zebularine, have been proven to suppress cellular growth, arrest cell cycle, and induce cell apoptosis of various tumor cells [8,9]. For example, Chen et al. reported that the growth of spontaneous mammary tumors was inhibited by zebularine treatment, which caused early onset of tumor cell necrosis and apoptosis in mice [10]. However, high side effects and toxicity are serious concerns. Compared with these chemical compounds, the use of natural products for cancer therapy might have some advantages, as they have low toxicity, minor side-effects, and are cost effective.

Green tea, a popular natural beverage worldwide, has many physiological and pharmacological benefits. Mounting evidence has shown that (−)-epigallocatechin-3-gallate (EGCG), the major chemical ingredient of green tea, can prevent carcinogenesis by inducing cancer cell apoptosis or arresting the cell cycle. In addition, EGCG has antioxidant, anti-inflammatory, antiproliferative, anti-angiogenic, and antimetastatic effects [11,12]. Fang et al. [13] reported that EGCG not only inhibited proliferation, invasion, and tumor growth, but also suppressed gelatinase activity by upregulation of E-cadherin and β-catenin, which induced apoptosis of nasopharyngeal carcinoma cells. EGCG also has been found to inhibit cell proliferation and migratory behavior of triple negative breast cancer cells [14]. The in vivo experiment found that an oral drug delivery system with EGCG presented a remarkable antiproliferative effect on breast tumor–bearing mice [15]. However, the regulatory target and molecular mechanism of EGCG action need to be further explicated in breast cancer. Recent investigations suggest that EGCG may prevent carcinogenesis by multiple epigenetic processes, including DNA methylation and histone acetylation [16,17]. A previous study reported that the expression of signal peptide-CUB (complement protein C1r/C1s, Uegf, and Bmp1)-EGF (epidermal growth factor) domain-containing protein 2 (*SCUBE2*) gene was significantly inhibited in breast cancer cells [18]. In the preliminary experiment of this study, we found that the promoter region of *SCUBE2* was hypermethylated in breast tumor. Therefore, we hypothesized that EGCG may play important roles in regulating the methylation and expression of the tumor suppressor gene *SCUBE2* and further suppress migration and invasion of breast cancer cells. The objective of this study was to clarify the epigenetic mechanism of the EGCG molecule and provide a scientific basis for the further application of EGCG in the treatment of human breast cancer.

## 2. Results

### 2.1. Effect of EGCG on the Growth and Viability of Human Breast Cancer Cells

**Figure 1:**
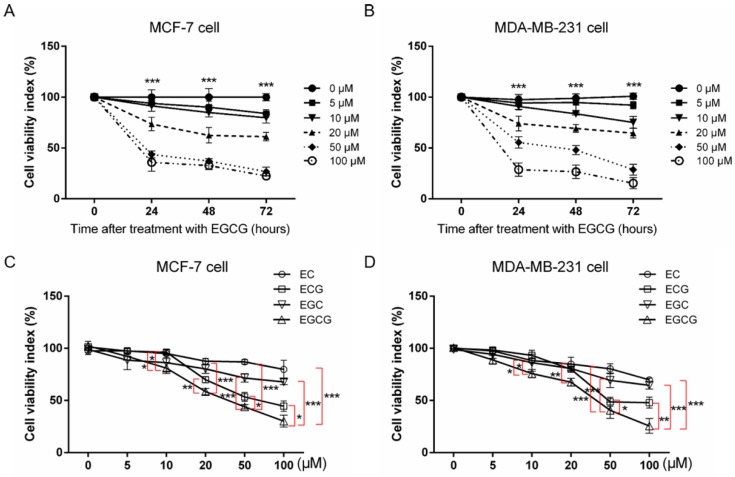
(−)-Epigallocatechin-3-gallate (EGCG) inhibits cell viability of breast cancer cells. (**A**) Cell viability of the MCF-7 cell line treated with various EGCG doses for 24, 48, and 72 h. (**B**) Cell viability of the MDA-MB-231 cell line treated with various EGCG doses for 24, 48, and 72 h. We compared the cell viabilities of MCF-7 or MDA-MB-231 cells treated with various EGCG concentrations at 24, 48, and 72 h (one-way ANOVA; *** *p* < 0.001). (**C**) Cell viability of MCF-7 treated with various concentrations of EC, epicatechin; ECG, epicatechin gallate; EGC, epigallocatechin; and EGCG for 24 h. (**D**) Cell viability of MDA-MB-231 treated with various concentrations of EC, ECG, EGC, and EGCG for 24 h. We compared the cell viabilities of MCF-7 or MDA-MB-231 cells treated with EC, ECG, EGC, and EGCG (one-way ANOVA followed by Dunn’s multiple comparison post hoc test; * *p* < 0.05, ** *p* < 0.01, and *** *p* < 0.001 the EC, ECG, EGC group versus EGCG group, respectively). Data were presented as mean ± SEM from six independent experiments.

We initially investigated the effect of EGCG on the breast cancer cell growth and viability. The MCF-7 and MDA-MB-231 cell lines were respectively treated with graded concentrations of EGCG for 24, 48, and 72 h, and then the cell viability was detected by MTT assay. As shown in Figure 1A,B, MCF-7 and MDA-MB-231 cell viability was significantly inhibited in response to EGCG treatment in a dose- and time-dependent manner. In addition, in order to determine the pharmacological and biological characteristics of EGCG, the IC_50_ values were calculated in breast cancer cells. In detail, the IC_50_ values were 54.25 ± 7.15, 40.35 ± 5.54, and 27.53 ± 1.02 μM in MCF-7 cells and 54.07 ± 9.52, 44.03 ± 3.61, and 27.12 ± 0.41 μM in MDA-MB-231 cells for 24, 48, and 72 h, respectively. In light of the concentration and time analysis, the EGCG dose of 20 μM and incubation time of 24 h was carried out in the subsequent series of experiments. To evaluate the pronounced effect of EGCG of green tea, we selected other three representative tea polyphenols (EC, epicatechin; ECG, epicatechin gallate; EGC, epigallocatechin) to compare their biological activity with that of EGCG, and found that EC, ECG, and EGC had slightly inhibitory effects on MCF-7 and MDA-MB-231 cell viability comparing to EGCG (Figure 1C,D). These results suggested that EGCG exhibited the potent function of growth inhibition.

### 2.2. EGCG Inhibits Lipopolysaccharide (LPS)-Mediated Induction of Cell Migration and Invasion

**Figure 2:**
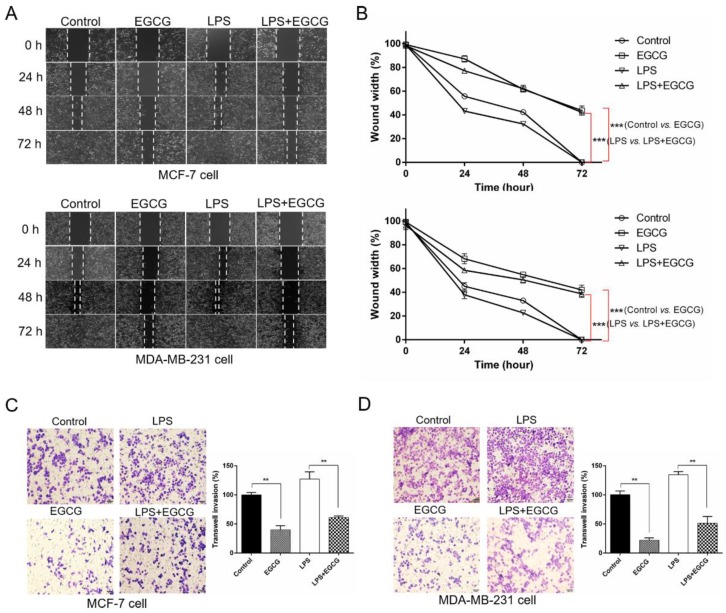
EGCG suppresses the migration and invasion of breast cancer cells. (**A**) Wound healing assay of MCF-7 (upper panel) and MDA-MB-231 (lower panel) cells treated with EGCG (20 μM), LPS (1 μg/mL), and combined EGCG (20 μM) and LPS (1 μg/mL). Images were taken before (0 h) and after wound closure (24, 48, and 72 h). (**B**) The data are shown as the percentage of the unhealed width by normalizing the width of the initial wound at 0 h (set to 100%). The significant differences were determined by the one-way ANOVA for area under the curve of each treatment (one-way ANOVA; *** *p* < 0.001 control vs. EGCG, *** *p* < 0.001 LPS vs. LPS + EGCG) (*n* = 3). MCF-7 cells (**C**) were treated as in (**A**) for 12 h and MDA-MB-231 cells (**D**) were treated as in (**A**) for 16 h, and then assayed for invasiveness. Columns in the graph (**C**,**D**) represented the analysis of the invasive cell count (one-way ANOVA; ** *p* < 0.01 control vs. EGCG, ** *p* < 0.01 LPS vs. LPS + EGCG). Scale bars, 50 μm. Data are shown as mean  ±  SEM from three independent experiments.

Given the significance of cell migration and invasion in regulating the process of forming distant metastasis, and a previous study suggesting that LPS (5 μg/mL) could trigger the action [19], we investigated the effect of EGCG on LPS-induced cell morphology changes of human breast cancer cells by the means of in vitro scratch wound healing and Transwell assays. MCF-7 and MDA-MB-231 cells were treated with EGCG, LPS, and LPS plus EGCG for 0~72 h. Results of the wound healing assay are shown in Figure 2A,B. The width ratios of cell-free areas were significantly higher in EGCG-treated MCF-7 cells compared with the control group at 24, 48, and 72 h (*p* < 0.001). Moreover, the combined treatment with LPS and EGCG demonstrated interactional antimigratory function compared to LPS-treated cells (*p* < 0.001). In addition, 42.2% and 38.8% wound width were still observed in MDA-MB-231 cells treated with EGCG or a combination of LPS plus EGCG for 72 h after scratching, respectively (Figure 2A,B). The Transwell assay also indicated that the number of invasive MCF-7 and MDA-MB-231 cells treated with EGCG or combined LPS and EGCG was significantly reduced compared to the untreated or LPS-treated cells (Figure 2C,D) (*p* < 0.01). These in vitro findings illustrated that EGCG can inhibit the migration and invasion of breast cancer cells.

### 2.3. EGCG Reverses SCUBE2 Loss of Expression in Human Breast Cancer Cells

**Figure 3:**
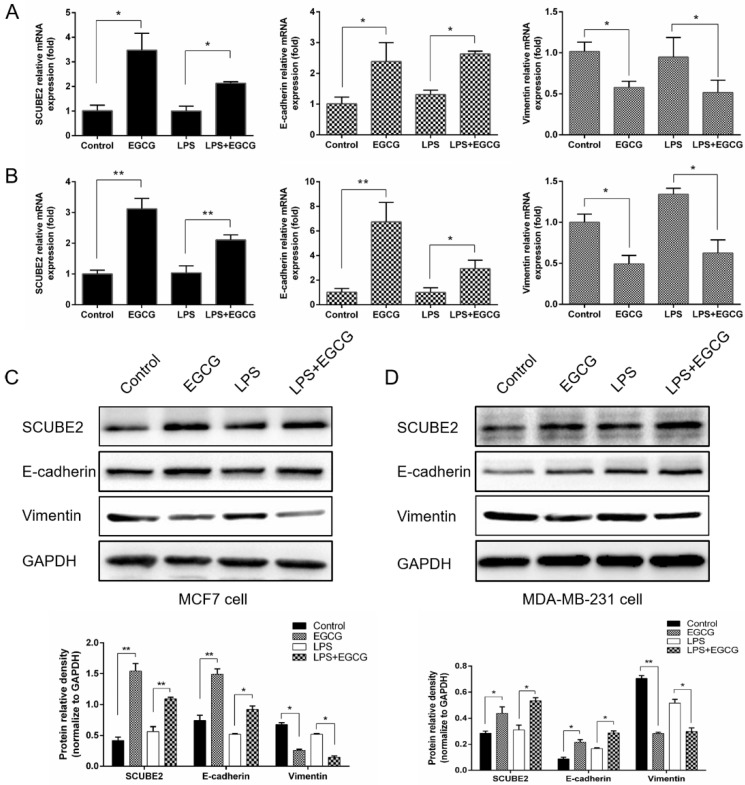
Effects of EGCG on the expression of *SCUBE2*, E-cadherin, and vimentin. MCF-7 and MDA-MB-231 cells were treated with EGCG (20 μM), LPS (1 μg/mL), and combined EGCG and LPS. The mRNA expression was determined by real-time PCR (**A**,**B**), and the protein levels were determined by Western blot (**C**,**D**). Statistical significance was determined by one-way ANOVA (* *p* < 0.05, ** *p* < 0.01). Densitometry analysis of relative *SCUBE2*, E-cadherin, and vimentin protein levels compared with glyceraldehyde 3-phosphate dehydrogenase (GAPDH) was performed by ImageJ 1.44 software. Data are shown as mean  ±  SEM from three independent experiments.

Epithelial–mesenchymal transition (EMT) plays an important role in cancer cell invasion and metastasis, and E-cadherin and vimentin are critical regulators of EMT. In this study, we revealed that EGCG treatment significantly increased E-cadherin and suppressed vimentin expression at both mRNA (Figure 3A,B) and protein level (Figure 3C,D) in MCF-7 and MDA-MB-231 cells. Additionally, the expression of E-cadherin significantly upregulated, while the vimentin was substantially decreased in the combination of LPS and EGCG treatment compared to the individual LPS treatment, which suggests that EGCG possesses an inhibitory effect on the EMT-related genes. A previous study reported that *SCUBE2* inhibited the migration and invasion of breast cancer cells by EMT reversal [18]. To determine whether *SCUBE2* participated in the EGCG inhibitory function, we also detected the *SCUBE2* expression in EGCG-treated cells. Initially, we found that *SCUBE2* showed a significant lower expression in breast cancer tissues than in the adjacent normal ones (Supplementary Figure S1). However, when breast cancer cells were treated with EGCG, the loss of *SCUBE2* expression status was reversed, causing higher *SCUBE2* levels compared to untreated cells. These results further implied that *SCUBE2* reactivation could be considered as a critical event in EGCG-mediated inhibitory effect.

### 2.4. Knockdown of Endogenous SCUBE2 Blocks the Inhibitory Effect of EGCG on Breast Cancer Cells

**Figure 4:**
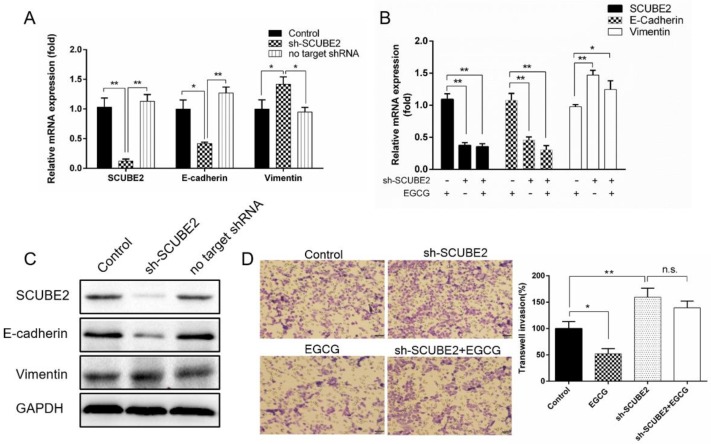
*SCUBE2* silencing affected the gene expression and cell invasion in MCF-7 cells. (**A**) The mRNA expressions of *SCUBE2*, E-cadherin, and vimentin were detected in the *SCUBE2* silencing cells (one-way ANOVA; * *p* < 0.05, ** *p* < 0.01 vs. sh-*SCUBE2* group). (**B**) The mRNA expressions of *SCUBE2*, E-cadherin, and vimentin were detected in the *SCUBE2* silencing cells that were treated with EGCG (one-way ANOVA; * *p* < 0.05, ** *p* < 0.01 vs. EGCG treatment group). (**C**) The protein levels of *SCUBE2*, E-cadherin, and vimentin were detected in the *SCUBE2* silencing cells. (**D**) Cell invasion was detected in the *SCUBE2* silencing cells that were treated with EGCG (one-way ANOVA; * *p* < 0.05, ** *p* < 0.01 vs. control). Scale bars, 50 μm. Data are expressed as mean ± SEM of three independent experiments. n.s. represents no significance.

We next determined whether *SCUBE2* silence can block the regulation of EGCG in the invasive behavior of breast cancer cells. The *SCUBE2* gene was specifically interfered with shRNA against the *SCUBE2* coding sequence in MCF-7 and MDA-MB-231 cells. The effectiveness of *SCUBE2* silencing reached up to 80% in comparison with the control group. We also found that the dominant silencing *SCUBE2* caused reduced E-cadherin levels and an increase in the expression of vimentin (Figure 4A,C and Supplementary Figure S2). However, when the *SCUBE2* knockdown cells were treated with EGCG, the E-cadherin and vimentin expressions were not significantly changed compared to those in the untreated cells (Figure 4B and Supplementary Figure S2). In addition, an in vitro invasion assay revealed that cells treated with EGCG showed inhibitory invasion, whereas the *SCUBE2* silencing cells showed marked 159% invasion, and the number of invasive cells did not present significant difference compared to the *SCUBE2* silencing cells treated with EGCG (Figure 4D and Supplementary Figure S2). These data indicate that EGCG confers anti-invasive properties in breast cancer cells via a *SCUBE2*-mediated pathway.

### 2.5. EGCG Reactivates SCUBE2 Expression by Reducing DNA Methylation and DNMT Activity

**Figure 5:**
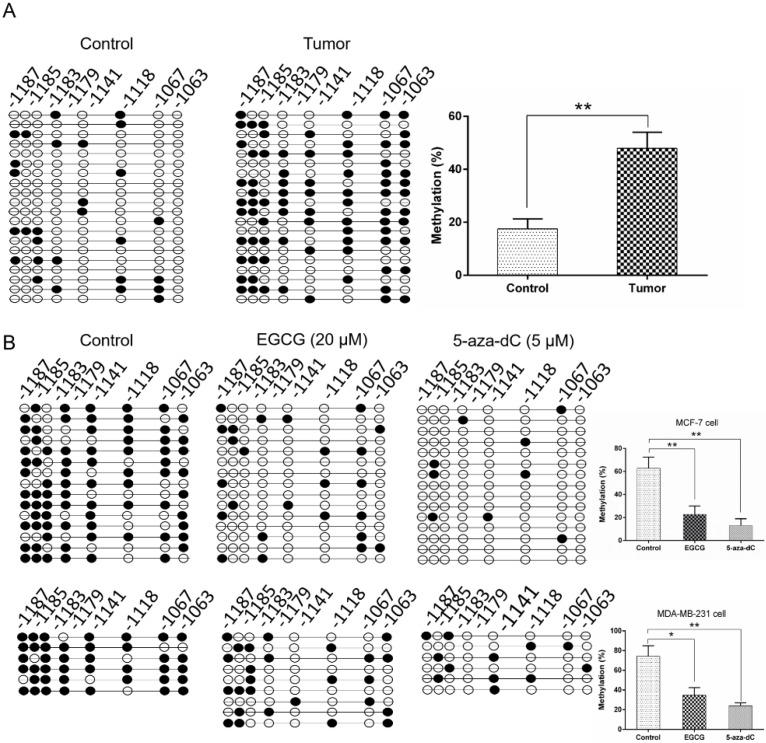
EGCG reduced the DNA methylation of the *SCUBE2* gene. (**A**) The *SCUBE2* methylation status in breast tumor tissue and adjacent control (Student’s *t*-test; ** *p* < 0.01 vs. control). (**B**) The methylation of the *SCUBE2* gene in MCF-7 (upper panel) and MDA-MB-231 cells (lower panel) treated with EGCG (one-way ANOVA; * *p* < 0.05, ** *p* < 0.01 vs. control). Data are shown as mean ± SEM from three independent experiments.

**Figure 6:**
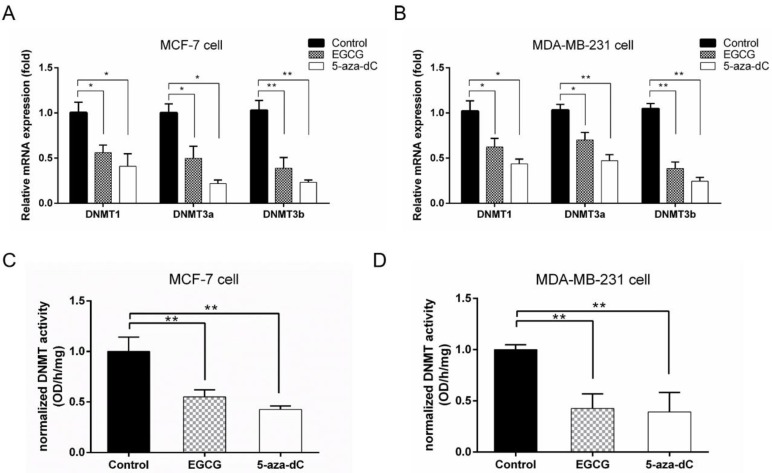
EGCG treatment decreased DNA methyltransferase (DNMT) expression and activity in breast cancer cells. The mRNA level of DNMT1, DNMT3a, and DNMT3b in MCF-7 (**A**) and MDA-MB-231 cells (**B**) treated with 20 μM EGCG or 5 μM 5-aza-dC. The results are presented as the expression of the individual mRNA with normalization to GAPDH (one-way ANOVA; * *p* < 0.05, ** *p* < 0.01 vs. control). (**C**) Normalized DNMT activity following 20 μM EGCG or 5 μM 5-aza-dC treatment in MCF-7 cells. (**D**) Normalized DNMT activity following 20 μM EGCG or 5 μM 5-aza-dC treatment in MDA-MB-231 cells. DNMT activity was significantly decreased with EGCG or 5-aza-dC treatment. The data (**C**,**D**) are normalized to the average values of control, untreated samples run within the same experiment, thus data were expressed relative to 1 (control) (one-way ANOVA; * *p* < 0.05, ** *p* < 0.01 vs. control). Data are expressed as mean ± SEM of three independent experiments.

Previous studies reported that EGCG had epigenetic effects on gene expression through predominantly regulating the DNA methylation levels [20]. The *SCUBE2* promoter contains several CpG islands that are preferential sites for methylation regulation. In this study, we found that the *SCUBE2* promoter in breast cancer tissues was significantly higher methylated in comparison to the adjacent control (*p* < 0.01) (Figure 5A). Importantly, as shown in Figure 5B, the *SCUBE2* promoter presented significantly decreased methylation in response to EGCG or 5-aza-dC treatment of MCF-7 and MDA-MB-231 cells (*p* < 0.05 or *p* < 0.01). Notably, 5-aza-dC, as a positive control, could significantly decrease the level of global DNA methylation. In addition, EGCG and 5-aza-dC also had similar effects on EMT-associated gene expression, migration, and invasion of breast cancer cells (Supplementary Figure S3). Given that DNMTs catalyzed the process of DNA methylation, we detected the mRNA expression of the DNMTs using real-time PCR (Figure 6A,B). The mRNA levels of DNMT1, DNMT3a, and DNMT3b were significantly decreased (*p* < 0.05 to *p* < 0.01) in MCF-7 and MDA-MB-231 cells treated with EGCG or 5-aza-dC compared with those in untreated cells. The decrease in DNMT expression may result in reduced DNMT activity. Thus, the activity of DNMT was determined in MCF-7 and MDA-MB-231 cells after treatment. Consistently, a significantly marked decrease of total DNMTs activity was detected in MCF-7 and MDA-MB-231 cells treated with EGCG or 5-aza-dC (*p* < 0.01) (Figure 6C,D), which suggested that EGCG can reactivate the *SCUBE2* gene in human breast cancer cells by reducing DNMT activity and DNA methylation.

## 3. Discussion

In recent years, extensive studies have reported that epigenetic modifications play crucial roles in the cancer physiology and etiology [21,22,23]. DNA methylation, one of the most important epigenetic events, especially contributes to the regulation of functional gene expression and phenotypic alterations. Aberrant genetic methylation often associates with the silencing expression of tumor suppressor genes, which could be considered as a hallmark of cancer and subsequently results in tumor development and progression [24]. Polyphenols of green tea have been proven to possess multiple anticarcinogenic activities in various human cancers, such as breast cancer by in vitro and in vivo experiments [25,26,27,28,29]. Epigenetic regulations, such as DNA methylation, histone acetylation, and noncoding RNA modifications, could respond to the dietary tea polyphenols and then regulate cellular process and modify the risk of cancer. A previous study reported that a reduced methylome status was associated with the intake of green tea in breast cancer patients [30], but the precise molecular mechanisms need to be fully elucidated. In this study, we investigated the phenotypic effect of tea catechins on human breast cancer cells and clarified the epigenetic mechanism of how EGCG regulated the DNA methylation of the *SCUBE2* gene.

The active ingredients in green tea are polyphenolic compounds, known as catechins, including EGCG, EC, ECG, and EGC. Among them, EGCG is the most abundant catechin accounting for 50% of the total amount [31]. Previous study has reported that EGCG was responsible for the beneficial bioactivities of green tea and contributed to various biological processes, including anti-inflammatory, antiviral, antioxidative, and antitumor abilities [32,33,34,35]. In addition, EGCG was validated to possess the therapeutic effects in clinical and animal studies [36,37]. In this study, we have characterized that EGCG significantly inhibits the growth of MCF-7 and MDA-MB-231 cells, while EC, ECG, and EGC are weaker modulators of cell viability. Consistently, Du et al. [38] determined the effects of different polyphenol compounds on human colorectal cancer cells, and found that EGCG showed the most potent antiproliferative effects, and significantly induced cell cycle arrest in the G1 phase and cell apoptosis compared to other polyphenols. Pathological studies reported that EGCG had the inhibitory effects on the histological severity of lesions and tumor progression in various cancer types [39,40]. Our study revealed that EGCG significantly suppressed migration and invasion of the human breast cancer cells, and the regulatory role was dose and time dependent, which may provide a promising rationale for the clinical application of EGCG as a breast cancer chemotherapy drug.

Given that epigenetic abnormalities greatly contributed to the tumorigenesis and progression, the rapid growing field of epigenetic drug exploration has immediately gained traction largely in cancer for therapeutic purposes. For instance, decitabine and 5-azacytidine, used as DNMT inhibitors, have been clinically used in the therapy of high-risk myelodysplastic syndrome; however, these agents exhibited extensive toxicity and may destroy the functional and structural patterns of normal cells and even cause canceration [41]. EGCG is considered to be a natural and nontoxic agent for targeting the tumor epigenome. Previous studies have reported that EGCG, used as an epigenetic modifier, epigenetically reversed the methylation and expression of silenced genes such as retinoic acid receptor β (*RAR*β), *O*^6^-methylguanine methyltransferase (*MGMT*)*,* and death-associated protein kinase-1 (*DAPK1*) by downregulating the DNMT activity in cancer cells [20,42]. In this study, we proved that MCF-7 and MDA-MB-231 cells incubated with EGCG presented significantly reduced DNMT activity compared to the untreated cells. Tyagi et al. [8] revealed that co-treatment with EGCG and 5-aza-dC showed significantly greater inhibition of growth of breast cancer cells compared to individual treatments and presented no significant toxicity to normal cells. These results suggest that EGCG is promising as a supplementary potion that can alleviate the adverse reaction of epigenetic drugs.

The epigenetic modifications referring to heritable decoration of DNA, RNA, and histones could modulate the expression of functional genes independently of genic sequence alterations. *SCUBE2*, coding a secreted and cell-surface glycoprotein with multiple domains, has been reported to be a novel tumor suppressor gene in various human cancers including colorectal, endometrial, and breast cancer [43,44,45]. Previous study demonstrated that *SCUBE2* suppressed mobility and invasiveness of breast carcinoma cell by regulating the expression of EMT marker genes to increase the formation of the epithelial adherens junctions and drive the reversal of EMT [18]. Our immunohistochemical assay illustrated that the protein level of *SCUBE2* was significantly lower in tumor tissue than that in control. Further detection revealed that the *SCUBE2* gene promoter region was hypermethylated in the histologic breast tumor. Silencing *SCUBE2* triggered the acceleration of the E-cadherin and the decrease of the vimentin in breast cancer cells. Correspondingly, Lin et al. [46] reported that overexpression of *SCUBE2* inhibited cell proliferation and reduced the tumor growth of the MCF-7 xenograft nude mice. In the present study, we identified for the first time that EGCG treatment reactivated the *SCUBE2* expression by reducing the methylation status, finally leading to the inhibition of breast cancer progression. However, when EGCG was applied in the *SCUBE2* knock-down cells, the migration and invasion of breast cancer cells was remarkably alleviated. These results suggested that *SCUBE2* may act as a causative epigenetic target for the EGCG pharmaceutical therapy in tumor development.

In conclusion, this study for the first time reported that EGCG can reverse the DNA methylation status and reactivate the expression of the *SCUBE2* gene by reducing DNMT expression and activity in human breast cancer cells, MCF-7 and MDA-MB-231. Our research revealed that the alterations of epigenetic modifications within the functional genes responding to EGCG treatment were considered to be the molecular basis for breast cancer chemoprevention. This study is of importance for understanding the regulatory molecular mechanisms of EGCG and providing extensive evidences for further clinical applications of EGCG against human cancers. However, further research is needed to verify the chemopreventive mechanisms of EGCG in nontumor breast cell lines, animal models, and the clinical trials of breast cancer patients, as well as to determine the epigenetic events, including DNA methylation and other histone modifications. Cumulatively, this study revealed the epigenetic roles of EGCG in regulating *SCUBE2* methylation, which provided promising scientific proof for clinical efficacy of EGCG and the exploration of a novel epigenetic drug.

## 4. Material and Methods

### 4.1. Cell Culture and Treatment

Human breast cancer cell lines MCF-7 (cat. no. HTB-22) and MDA-MB 231 (cat. no. HTB-26) were purchased from ATCC (American Type Culture Collection). Cells were maintained in Dulbecco’s modified Eagle’s medium (DMEM) (cat. no. 10569-010) supplemented with 10% fetal bovine serum (cat. no. 10099-141; both Gibco; Thermo Fisher Scientific, Inc., Waltham, MA, USA), 50 U/mL penicillin, and 50 mg/mL streptomycin in a humid atmosphere containing 5% CO_2_ at 37 °C. Cells were treated with either EGCG (20 μM) (cat. no. E4143-50MG), LPS (1 μg/mL) (cat. no. L3012-5MG), 5-aza-dC (5 μM) (cat. no. A3656-5MG; all Sigma-Aldrich; MERCK, Inc., St. Louis, MO, USA), or a combination of EGCG and LPS for indicated time period. For combined treatment with EGCG and LPS, cells were pretreated with LPS for 1 h followed by the treatment with EGCG. Every alternate day, the culture medium was replaced with the fresh media and EGCG was added with a fresh dose.

### 4.2. MTT Assay

A methyl thiazolyl tetrazolium (MTT) assay was performed to evaluate the effects of EGCG treatment on cell viability. Cells were seeded in 96-well plate at a density of 5 × 10^3^ cells in each well. After 24 h of seeding, cells were incubated with different concentrations of EGCG: 0, 5, 10, 20, 50, and 100 μM. After 24, 48, and 72 h, respectively, medium was removed, cells were washed with 1 × phosphate buffer saline (PBS), and then the MTT (1 mg/mL) (cat. no. M2128-100MG; all Sigma-Aldrich; MERCK, Inc., Kenilworth, NJ, USA) was added to each well and incubated at 37 °C for 4 h. Media were removed, dimethyl sulfoxide (DMSO, 150 μL/well) was used to dissolve formazan. Absorbance values were measured at 570 and 650 nm (as background) on a microplate reader (Molecular Devices, Inc., San Jose, CA, USA). Each treatment was run in sextuplicate and the experiment was repeated twice.

### 4.3. Wound Healing Assay

MCF-7 and MDA-MB 231 cells were seeded at a density of 2 × 10^5^ cells in each well of the six-well plates and allowed to adhere overnight. When the cells grew at 90% confluence, cell monolayer was scratched using a sterile pipette tip, and three scratch wounds were made in each well. The scratched cells were washed three times with 1 × PBS to remove the nonadherent cells. According to different treatments, the wells were divided into four groups: Blank (grow medium), LPS (grow medium with 1 μg/mL LPS), EGCG (grow medium with 20 μM EGCG), and LPS plus EGCG (pretreated with 1 μg/mL LPS for 1 h and added 20 μM EGCG). The cells in the four groups were incubated at 37 °C/5% CO_2_-humidified incubator and photographed at time points (0, 24, 48, and 72 h). Cell migration areas were calculated using Image-Pro Plus software (version 6.0, Media Cybernetics, Inc., Rockville, MD, USA).

### 4.4. Transwell Invasion Assay

The invasiveness of MCF-7 and MDA-MB 231 cells was tested using Transwell chambers (Corning Incorporated, Corning, NY, USA). The upper chambers of Matrigel (50 μL; BD Biosciences, San Jose, CA, USA)-coated inserts were seeded with 1 × 10^4^ cells for the invasion assay. Cells in the upper chambers were cultured with the following mediums: DMEM, DMEM with 1 μg/mL LPS, DMEM with 20 μM EGCG, and DMEM with 1 μg/mL LPS and 20 μM EGCG (pretreated with LPS for 1 h and added EGCG), respectively. Lower chambers were added with regular complete culture medium as chemoattractant. After incubating for 24 h, the noninvasive cells were slightly removed from the upper surface using a cotton swab, and the invaded cells were fixed in 4% paraformaldehyde and stained with the crystal violet (0.1%). Invaded cells were analyzed under a microscope at a ×200 magnification. Each sample was assayed in triplicate.

### 4.5. RNA Extraction and Real-time PCR

Total RNA from cells was isolated using TRIzol reagent (Invitrogen; Thermo Fisher Scientific, Inc.). The first-strand cDNA was synthesized using a PrimeScript RT Reagent Kit with gDNA Eraser (Perfect Real Time) (cat. no. RR047A; Takara Bio, Inc., Otsu, Japan) with 2 μg total RNA as the template. All primer sequences for qPCR are shown in Supplementary Table S1. The qPCR experiments were performed using a SYBR Green kit (cat. no. 4367659; Invitrogen, Thermo Fisher Scientific, Inc., MA, USA) conducted on a CF X 96^TM^ RealTime Detection System (Bio-Rad Laboratories, Inc. Hercules, CA, USA). Glyceraldehyde 3-phosphate dehydrogenase (GAPDH) was used for sample normalization. Thermal cycling conditions were as follows: 95 °C for 30 s followed by 40 cycles of 95 °C for 10 s, 60 °C for 10 s, and 68 °C for 20 s. Each experiment was repeated in triplicate. The relative gene expression was normalized to the GAPDH expression level and the data were quantified using the 2^–ΔΔCq^ method [47].

### 4.6. Western Blot

Cells were lysed from both the control group and treated MCF-7 or MDA-MB 231 cells using radioimmunoprecipitation assay (RIPA) buffer (cat. no. PL007; Sangon Biotech Co., Ltd., Shanghai, China) for 20 min on ice. Protein concentrations were determined using the bicinchoninic acid (BCA) method (Bio-Rad Laboratories, Inc. Hercules, CA, USA) to make equal loading. Total proteins were resolved in the SDS–PAGE gel and then transferred to a PVDF membrane. The membrane was blocked with 5% skim milk in Tris saline with Tween (TBST) buffer for 2 h at room temperature, and then it was incubated overnight at 4 °C with primary antibodies that were specific for anti-*SCUBE2* (1:1000; cat. no. sc-398607; Santa Cruz, Inc., Dallas, TX, USA), anti-E-cadherin (1:1000; cat. no. #3195; Cell Signaling Technology, Inc., Danvers, MA, USA), anti-vimentin (1:1000; cat. no. #5741; Cell Signaling Technology, Inc., Danvers, MA, USA), and anti-GAPDH (1:2000; cat. no. sc-293335; Santa Cruz, Inc., Dallas, TX, USA). The hybridized membrane was washed three times with TBST buffer and incubated with horseradish peroxidase (HRP)-conjugated secondary antibody (1:5000; cat. no. 115-035-003; Jackson ImmunoResearch Laboratories, Inc.) for 2 h at room temperature. Finally, the membrane was rinsed with 1 × TBST five times, and the protein bands were visualized by treating with chemiluminescent HRP substrate (cat. no. WBKLS0500; Merck KGaA, Darmstadt, Germany). 

### 4.7. Silencing of SCUBE2 Expression with Small Hairpin RNA (shRNA)

The sequences for *SCUBE2* shRNA were: sense- CCGGCGGCTGCAAGAAA GGATTTAACTCGAGTTAAATCCTTTCTTGCAGCCGTTTTTGGTACCG and antisense AATTCGGTACCAAAAACGGCTGCAAGAAAGGATTTAACTCGAGTT AAATCCTTTCTTGCAGCCG. The shRNA sequences were linked to pLKO.1 vector (Sigma-Aldrich; MERCK, Inc. St. Louis, MO, USA) by *Age* I and *Eco*R I digesting sites. Along with packaging constructs pCMV-VSV-G and pCMV-Gag-Pol, 293T cells were transfected with pLKO.1-shRNA using lipid transfection Lipofectamine 3000 (Invitrogen; Thermo Fisher Scientific, Inc.). After 72 h of infection, the culture medium was concentrated, and the virus titer was determined using a Lenti-Pac^TM^ human immunodeficiency virus (HIV) qRT-PCR Titration Kit (cat. no. 812WY0201, GeneCopoeia, Inc.; San Diego, CA, USA). MCF-7 or MDA-MB-231 cells were infected with the virus (multiplicity of infection (MOI) = 5) and 8 μg/mL polybrene. After 48 h of infection, the previous media was replaced with fresh complete DMEM media. After 24 h, the infected cells were artificially selected by 2 μg/mL puromycin for two weeks. The surviving cells were tested for *SCUBE2* knockdown efficiency using qPCR and Western blot.

### 4.8. Immunohistochemistry of Human Breast Tissue

Human samples were collected from patients after surgery at Xinzhou People’s Hospital (Wuhan, China). A total of ten breast tumor samples and ten adjacent normal samples were collected from patients. All subjects gave their informed consent for inclusion before they participated in the study. The study was conducted in accordance with the Declaration of Helsinki, and the protocol was approved by the Ethics Committee of Xinzhou People’s Hospital (no. HCXZ-2017-6-025). 

The tissue sections were incubated for 45 min with primary antibody (anti-*SCUBE2*; 1:250; cat. no. sc-398607; Santa Cruz, Inc., Dallas, TX, USA), and the subsequent treatment was carried out using the immunohistochemistry kit (cat. no. KIT-5001; MaxVision, Fuzhou, China) according to the manufacturer’s instructions. PBS was used as the blank control for the specificity of the primary antibody. The sections were rinsed with 1 × PBS three times and probed with the immunoglobulin G (IgG) secondary antibodies (1:400; cat. no. 115-035-003; Jackson ImmunoResearch Laboratories, Inc., UT, USA) for 30 min at room temperature. Then, the sections were washed three times and stained with 3,3′-diaminobenzidine (cat. no. DA1010; Beijing Solarbio Science and Technology, Beijing, China) for 1 min at room temperature, and the samples were counterstained with hematoxylin (cat. no. 517-28-2; Beijing Solarbio Science and Technology) for 50 s at room temperature and then dehydrated with gradient concentrations of ethanol and xylene. Finally, the whole slides were coverslipped and digitally scanned with a BX53 microscopy (Olymp, Japan).

### 4.9. SCUBE2 Promoter Methylation

Whole genomic DNA was extracted from cells or tissues using a DNA isolation kit (cat. no. D1700; Solarbio, Beijing, China). The DNA was treated with the EZ-DNA methylation Gold Kit (cat. no. D5005; Zymo Research, Orange, CA, USA). The treated DNA was used as the template for a PCR reaction with the primer pair F- TTTTTTTGATTTTTAAAGGAATGGT and R- AAATCTTCCCATATCACACTTAACAC. The PCR products were purified and ligated to the pGEM-T plasmid (Promega, Madison, WI, USA). The constructed plasmid was transformed into JM101 competent cells (Promega, Madison, WI, USA) and cultured for 16 h. For each sample, a total of 30 clones were sequenced to evaluate the methylation state.

### 4.10. DNMT Activity Assay

After treating MCF7 and MDA-MB 231 cells with 20 μM EGCG or 5 μM 5-aza-dC for 48 h, the cell nuclear extracts were isolated using EpiQuik Nuclear Extraction Kit (cat. no. OP-0002-1, EpiGentek, NY, USA) according to the manufacturer’s instructions. Total DNMT activity in nuclear extracts was determined by EpiQuik DNMT Colorimetric activity/inhibition kit (cat. no. P-3009-48; EpiGentek, NY, USA). First, the test strip wells were coated with a unique cytosine-rich DNA substrate, and DNMT enzymes in the nuclear extracts could methylate the DNA substrate by transferring a methyl group to cytosine from AdoMet (S-adenosylmethionine). Then, methylated DNA was recognized by an anti-5-methylcytosine antibody. The amount of methylated DNA was proportional to the DNMT enzymatic activity, which was quantified at 450 nm in a plate reader. DNMT activity was calculated by the following equation:

DNMT Activity (OD/h/mg) = (sample OD − blank OD)/(protein amount (µg) ×  initial incubation time in hours) × 1000.(1)

The DNMT activity values in samples were normalized to values obtained from control (untreated cells), which were expressed relative to one within the same experiment.

### 4.11. Statistical Analysis

SPSS software (version 20.0, SPSS Inc., Chicago, IL, USA) was used for statistical analysis in this study. Data are shown as mean ± SEM of three separate experiments performed in triplicate. The significant differences were determined between groups by using a Student’s *t*-test or one-way Analysis of Variance (ANOVA) followed by post hoc Dunnett’s test. A *p*-value of less than 0.05 was considered to be significant.

## Supplementary Materials

The following are available online. Figure S1: *SCUBE2* possessed low expression in breast cancer tissue. Figure S2: *SCUBE2* silencing affected the gene expression and cell invasion in MDA-MB-231 cells. Figure S3: EGCG and 5-aza-dC have similar roles in regulating breast cancer cell activities. Table S1: Primer pairs used in this study.
