# Supplementary material for: The Inhibitory Effect of (−)-Epigallocatechin-3-Gallate on Breast Cancer Progression via Reducing SCUBE2 Methylation and DNMT Activity
Source: Molecules. 2019 Aug 9;24(16):2899. doi: 10.3390/molecules24162899 (PMC6719997; doi:10.3390/molecules24162899)
Supplement: Supplementary file 1 [file molecules-24-02899-s001.zip › Supplementary materials/Supplementary Figure S1.docx]

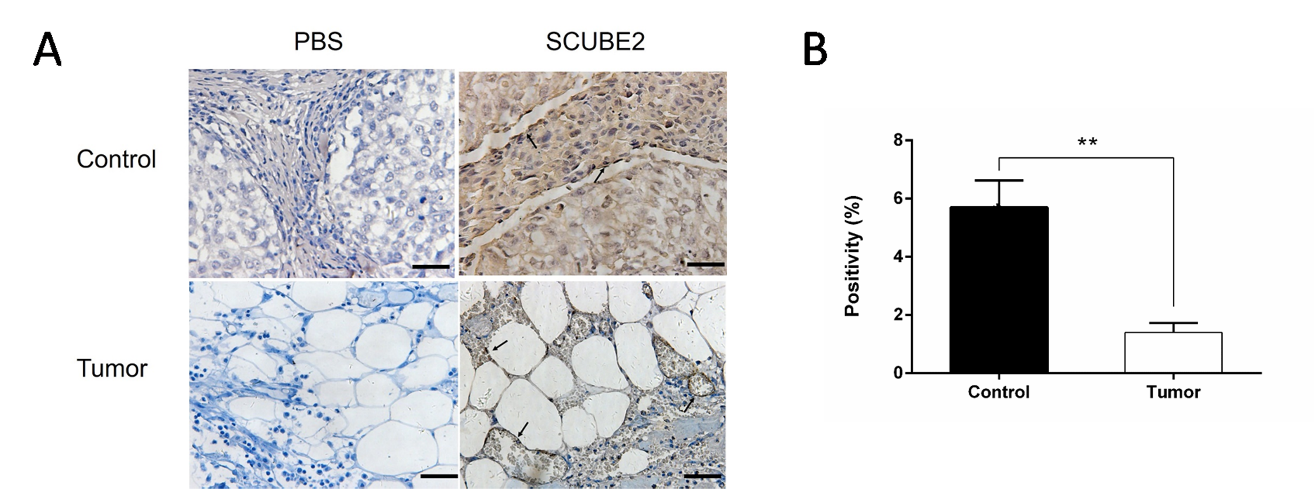


**Figure S1. SCUBE2 possessed low expression in breast cancer tissue.** (A) Immunohistochemistry of the SCUBE2 in breast tumor and control. (B) The quantization of the immunohistochemistry result. Data were expressed as mean ± SEM from ten tissue sections. Statistical significance was determined by Student’s *t*-test (***P*<0.01 vs. Control).
