# Supplementary material for: The Inhibitory Effect of (−)-Epigallocatechin-3-Gallate on Breast Cancer Progression via Reducing SCUBE2 Methylation and DNMT Activity
Source: Molecules. 2019 Aug 9;24(16):2899. doi: 10.3390/molecules24162899 (PMC6719997; doi:10.3390/molecules24162899)
Supplement: Supplementary file 1 [file molecules-24-02899-s001.zip › Supplementary materials/Supplementary Figure S2.docx]

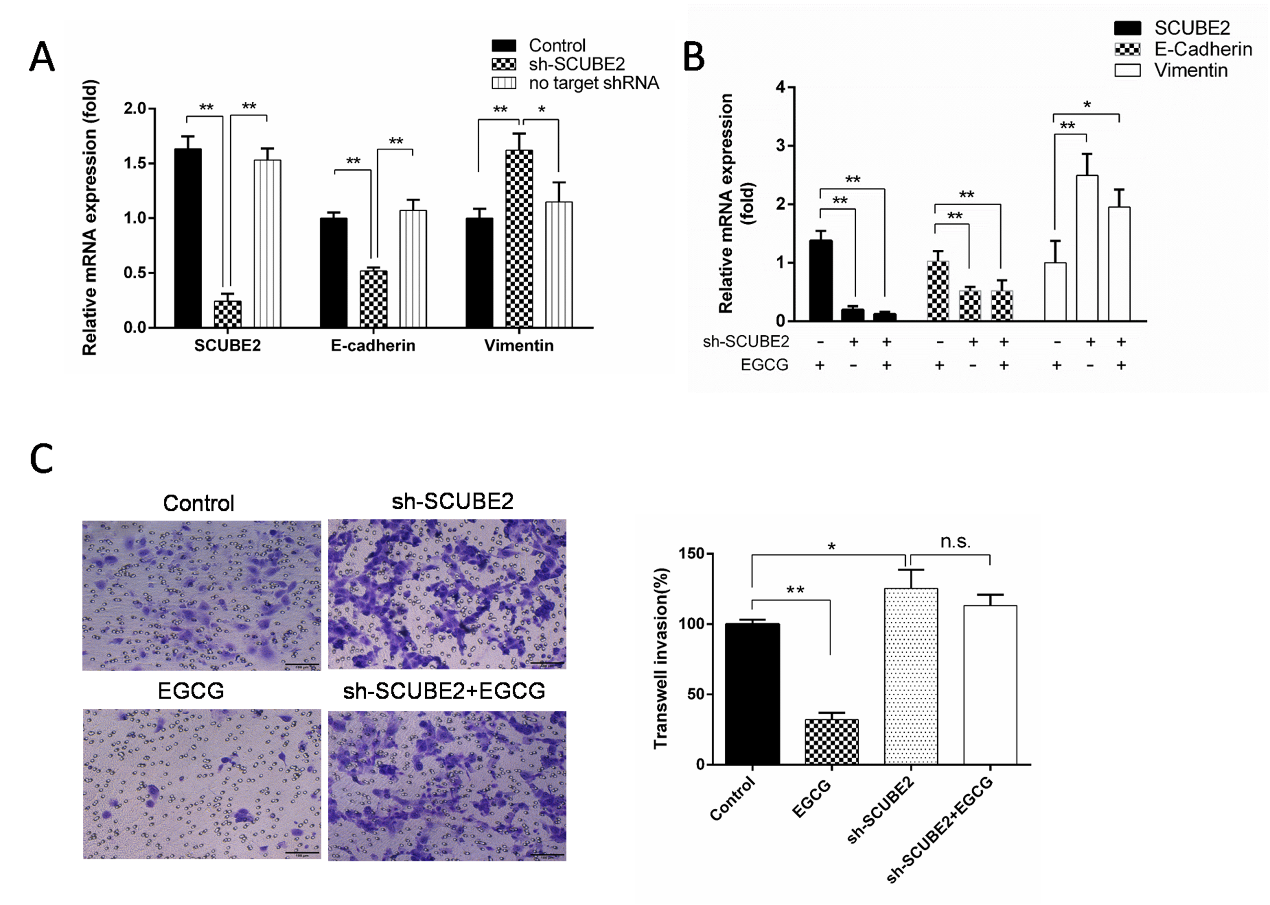


**Figure S2. SCUBE2 silencing affected the gene expression and cell invasion in MDA-MB-231 cells.** (A) The mRNA expression of the SCUBE2, E-cadherin, and vimentin were detected in the SCUBE2 silencing cells (One-way ANOVA; **P*<0.05, ***P*<0.01 vs. sh-SCUBE2 group). (B) The mRNA expression of the SCUBE2, E-cadherin, and vimentin was detected in the SCUBE2 silencing cells that were treated with EGCG (One-way ANOVA; **P*<0.05, ***P*<0.01 vs. EGCG treatment group). (C) Cell invasion was detected in the SCUBE2 silencing cells that were treated with EGCG (One-way ANOVA; **P*<0.05, ***P*<0.01 vs. Control). Scale bars, 50 μm. Data were expressed as mean ± SEM of three independent experiments. n.s. represented no significance.
