# Supplementary material for: The Inhibitory Effect of (−)-Epigallocatechin-3-Gallate on Breast Cancer Progression via Reducing SCUBE2 Methylation and DNMT Activity
Source: Molecules. 2019 Aug 9;24(16):2899. doi: 10.3390/molecules24162899 (PMC6719997; doi:10.3390/molecules24162899)
Supplement: Supplementary file 1 [file molecules-24-02899-s001.zip › Supplementary materials/Supplementary Figure S3.docx]

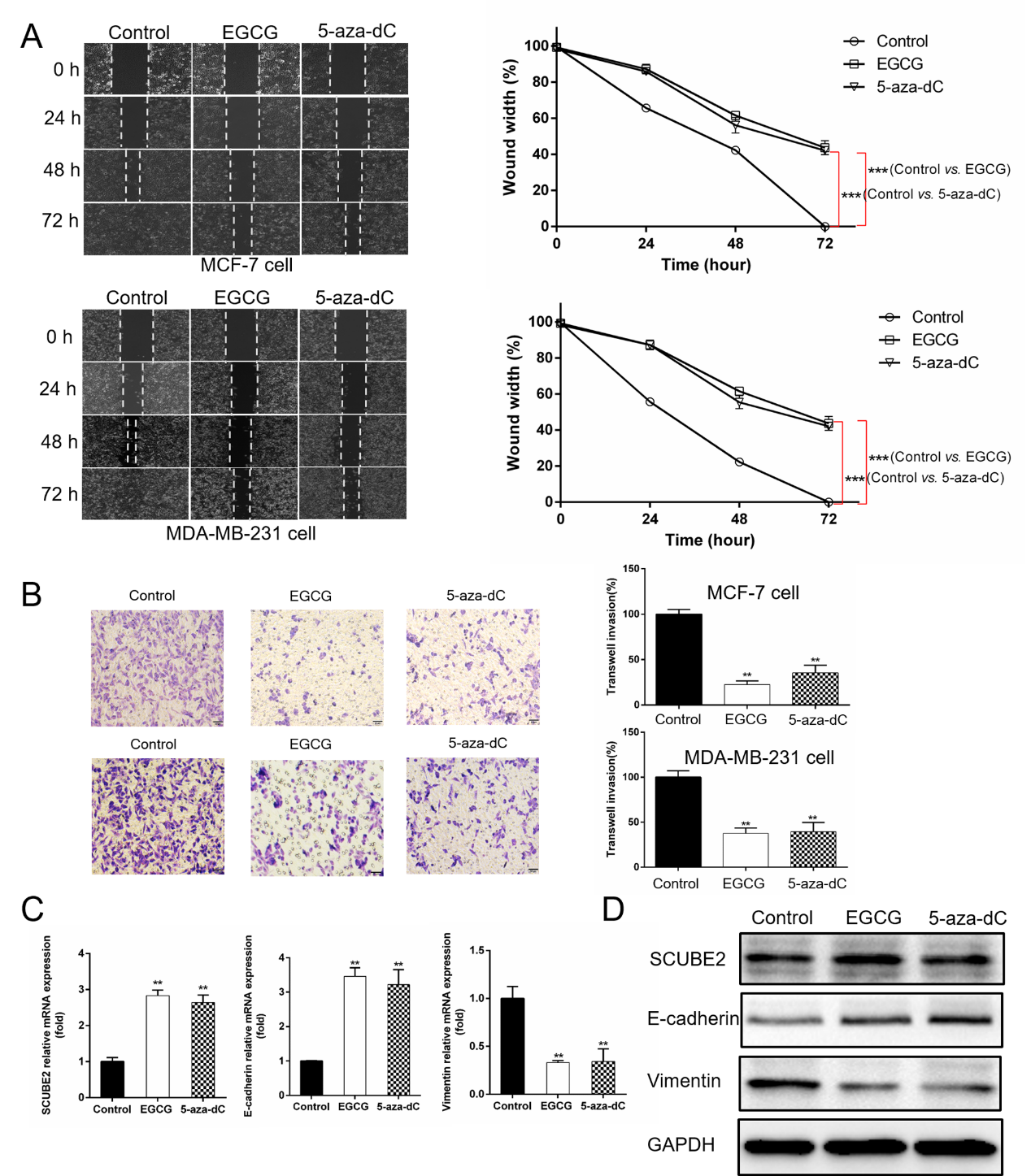


**Figure S3. EGCG and 5-aza-dC have similar roles in regulating breast cancer cell activities.** Cell migration (A) and invasion (B) were detected in MCF-7 and MDA-MB-231 cells treated with 20 μM EGCG or 5 μM 5-aza-dC (One-way ANOVA; ***P* < 0.01, ****P*<0.001 vs. Control) (n = 3). The significant differences in (A) were determined by the one-way ANOVA for area under the curve of each treatment. The mRNA (C) and protein levels (D) of the SCUBE2, E-cadherin, and vimentin were detected in MCF-7 cell treated with 20 μM EGCG or 5 μM 5-aza-dC (one-way ANOVA; ***P* < 0.01 vs. Control). Data were expressed as mean ± SEM of three independent experiments.
