# Supplementary material for: The Inhibitory Effect of (−)-Epigallocatechin-3-Gallate on Breast Cancer Progression via Reducing SCUBE2 Methylation and DNMT Activity
Source: Molecules. 2019 Aug 9;24(16):2899. doi: 10.3390/molecules24162899 (PMC6719997; doi:10.3390/molecules24162899)
Supplement: Supplementary file 1 [file molecules-24-02899-s001.zip › Supplementary materials/Supplementary Table S1.docx]

**Table S1.** Primer pairs used in this study.

| Primer name | Primer sequences (5’ to 3’) | Production  sizes (bp) | Tm  (^o^C) |
| --- | --- | --- | --- |
| qPCR primer for *SCUBE2* | F: CTGCCAGCATACCTGTGTCA | 107 | 60 |
|  | R: CCTCTTCCGAGCGGTGAAT |  |  |
| qPCR primer for *E-cadherin* | F: ACACCCGGGACAACGTTTAT | 115 | 60 |
|  | R: TTACGAGTCACTTCAGGCCG |  |  |
| qPCR primer for *Vimentin* | F: GCCCTTGACATTGAGATTGCC | 100 | 60 |
|  | R: CCCTCAGGTTCAGGGAGGAA |  |  |
| qPCR primer for *DNMT1* | F: TTATCCGAGGAGGGCTACCTG | 101 | 60 |
|  | R: CGTCCATTCACTTCCCGGTT |  |  |
| qPCR primer for *DNMT3a* | F: CAGTTGGCCCGGCAAAGAA | 110 | 60 |
|  | R: GCTGACGGAGGCTGGACT |  |  |
| qPCR primer for *DNMT3b* | F: GCACTACACAGACGTGTCCA | 108 | 60 |
|  | R: TAGTCCTTCAGAGGGGCGAA |  |  |
| qPCR primer for *GAPDH* | F: CCCACTCCTCCACCTTTGAC | 108 | 60 |
|  | R: CCACCACCCTGTTGCTGTAG |  |  |
| Primer for shRNA (SCUBE2) | F: CCGGCGGCTGCAAGAAAGGATTTAAC  TCGAGTTAAATCCTTTCTTGCAGCCGTTTTTGGTACCG | 64 | / |
|  | R: ATTCGGTACCAAAAACGGCTGCAAGAAAGGATTTA  ACTCGAGTTAAATCCTTTCTTGCAGCCG |  |  |
| Primer for methylation detection | F: TTTTTTTGATTTTTAAAGGAATGGT | 222 | 51 |
|  | R: AAATCTTCCCATATCACACTTAACAC |  |  |
